# Feasibility of a prototype newborn resuscitation monitor to study transition at birth, measuring heart rate and ventilator parameters, an animal experimental study

**DOI:** 10.1186/s13104-017-2530-z

**Published:** 2017-06-28

**Authors:** Jørgen E. Linde, Joar Eilevstjønn, Knut Øymar, Hege L. Ersdal

**Affiliations:** 1 Department of Pediatrics, Stavanger University Hospital, POB 8100, 4068 Stavanger, Norway; 2 Laerdal Medical and Laerdal Global Health, Stavanger, Norway; 3 Department of Clinical Science, University of Bergen, Bergen, Norway; 4 Department of Anesthesiology and Intensive Care, Stavanger University Hospital, Stavanger, Norway

**Keywords:** Newborn, Resuscitation, Monitoring

## Abstract

**Background:**

Every year, an estimated 10 million babies are born, non-breathing and in need of resuscitation. Advances in management have been made over the past decades, however, approximately 700.000 yearly deaths result from this global problem. A prototype newborn resuscitation monitor (NRM) (Laerdal Global Health, Stavanger, Norway) has been developed with the purpose of studying newborn resuscitation. The monitor has the ability to continuously display HR using dry electrode ECG technology, to measure tidal volume, pressure and end tidal CO_2_, and to store the results for later analysis. Such monitor could enhance the care providers performance, and hence survival of neonates, by displaying the quality and response of the given care. The aim of this preclinical study was to describe the abilities of the NRM to measure ventilation and heart rate parameters against pathophysiological responses to different induced conditions in a piglet i.e. increased deadspace, pressure and washout of surfactant.

**Methods:**

Piglets were chosen for the study, as they have tidal volumes of approximately 6 ml/kg, resembling the human neonate. Five piglets were anesthetized and intubated before starting positive pressure ventilation (PPV). The dry electrode ECG sensor of the NRM was placed over the abdomen, and experiments performed: (1) inducing different ventilation scenarios and (2) lavage of surfactant.

**Results:**

The NRM was capable of continuously displaying HR and detecting inflicted changes in ventilation and compliance of piglets. It could measure inflated and exhaled volume, the pressure of the ventilations and also the end tidal CO_2_.

**Conclusions:**

The NRM provides objective feedback in anesthetized animals, and may be used in clinical studies and hopefully generate new knowledge on neonatal transition and resuscitation. The monitor may be further developed for use in both low and high-resource settings.

## Background

Every year, an estimated 10 million babies are born non-breathing and in need of resuscitation [1]. Advances in management of newborn infants requiring delivery room resuscitation have been made over the past decades, however, approximately 700.000 yearly deaths result from this global problem [2]. In 2010, the International Liaison Committee on Resuscitation (ILCOR) raised the important question of whether improved monitoring of the newborn would be of benefit in facilitating effective resuscitation including positive pressure ventilation [2]. During positive pressure ventilation, pressure and volume may individually play important roles in the establishment of effective or harmful ventilations. Experimental data indicate that the ventilation volume does not correlate well with the peak ventilation pressure, and that the provider cannot correctly estimate the volume given when only pressure is displayed as feedback [3]. Moreover, a recent study has shown the value of end tidal carbon dioxide (EtCO_2_) as a useful measure of lung aeration and pulmonary blood flow [4].

Assessment of the heart rate (HR) is essential during neonatal resuscitation, as an indicator of effective ventilation and immediate feedback to the provider [5]. However, previous studies have shown that common methods for assessing HR are inaccurate, such as palpation of the umbilical cord and auscultation of the chest [6]. ECG seems to be the most reliable and accurate technology for immediate monitoring of the newborn HR and continuous feedback during resuscitation [7, 8]. Nevertheless, this technology is not commonly available in the delivery rooms. Monitors to guide ventilations are rarely available. A prototype newborn resuscitation monitor (NRM) (Laerdal Global Health, Stavanger, Norway) has been developed as a research tool with the purpose to study delivery room resuscitation and explore possibilities for monitoring resuscitation. However, there was a need to demonstrate the abilities of the monitor on live subjects in a controlled setting prior to the clinical studies on newborns. The NRM was constructed to record ventilation pressure and volume, hence compliance, as well as EtCO_2_ and HR during varying ventilation rates, inflating volumes and dead space. The monitor complies with current medical device technical standards, is CE-marked and approved for patient use.

Piglets were chosen for this purpose, partly because their required tidal volume approximates 6 ml/kg, resembling the human neonate [9].

The objective of this preclinical study was to demonstrate the functions of the NRM to record physiological changes in live subjects through exploring how (1) EtCO_2_ measurements were affected by changes in ventilation rate, inflated volume, and different dead space (simulating a face mask ventilation situation) and (2) lavage of surfactant affected the compliance and HR (simulating different clinical conditions in an infant).

## Methods

The study was performed at SEARCH (Sandnes Educational and Research Centre Høyland) animal laboratory in Sandnes, Norway. The protocol was approved by The Research Animal Council for the Rogaland Region. We included five piglets with postnatal age 1.5 (n = 1) and 2.5 weeks (n = 4), with median weight 5.1 kg (range 3.7–5.2 kg). As the monitor was CE marked and approved for use in humans, we chose a small sample size to test the functions and the signal data management before the clinical studies. The piglets were handled in accordance with the European Guidelines for Use of Experimental Animals by certified Federation of European Laboratory Animal Science Association category C researchers (FELASA C).

### Measurements

**Fig. 1:**
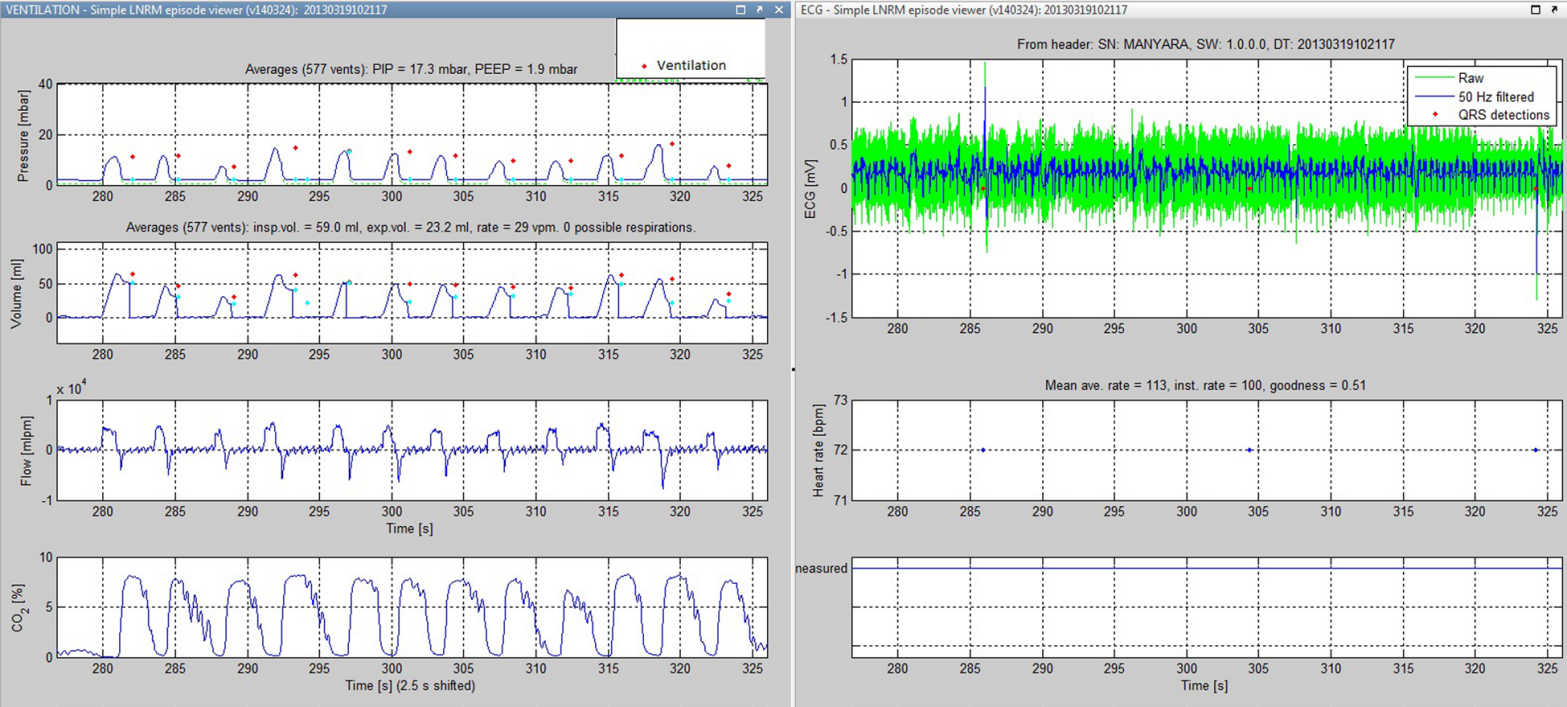
Recordings of ventilation and ECG signal data: examples of manual baseline ventilations and ECG from piglet B

The NRM has sensors capable of synchronously recording HR, airway pressure, flow, volume, and EtCO_2_. Flow and volume are measured using a flow sensor (MIM Gmbh, Krugzell, Germany) placed between the endotracheal tube (ETT) and the resuscitator bag. The flow sensor and its electronics are the same as used in the Florian respiration monitor (Acutronic AG, Zürich, Switzerland) that has previously been used in similar studies [10, 11]. The sensor has negligible flow resistance and dead space (1 ml), and measures airflow using hot wire anemometer technology. In addition, an airway adapter connecting two plastic tubes to the main cabinet is placed between the ETT and the bag. The first tube draws a sample of exhaled air (50 ml/min) for side stream CO_2_ measurement (ISA CO_2_ sensor, Masimo/PhaseIn AB,Danderyd, Sweden) (Fig. 1). The second tube measures airway pressure using a piezoresistive pressure sensor (MPXV5010, Freescale Semiconductor Inc, Austin, Tx).

**Fig. 2:**
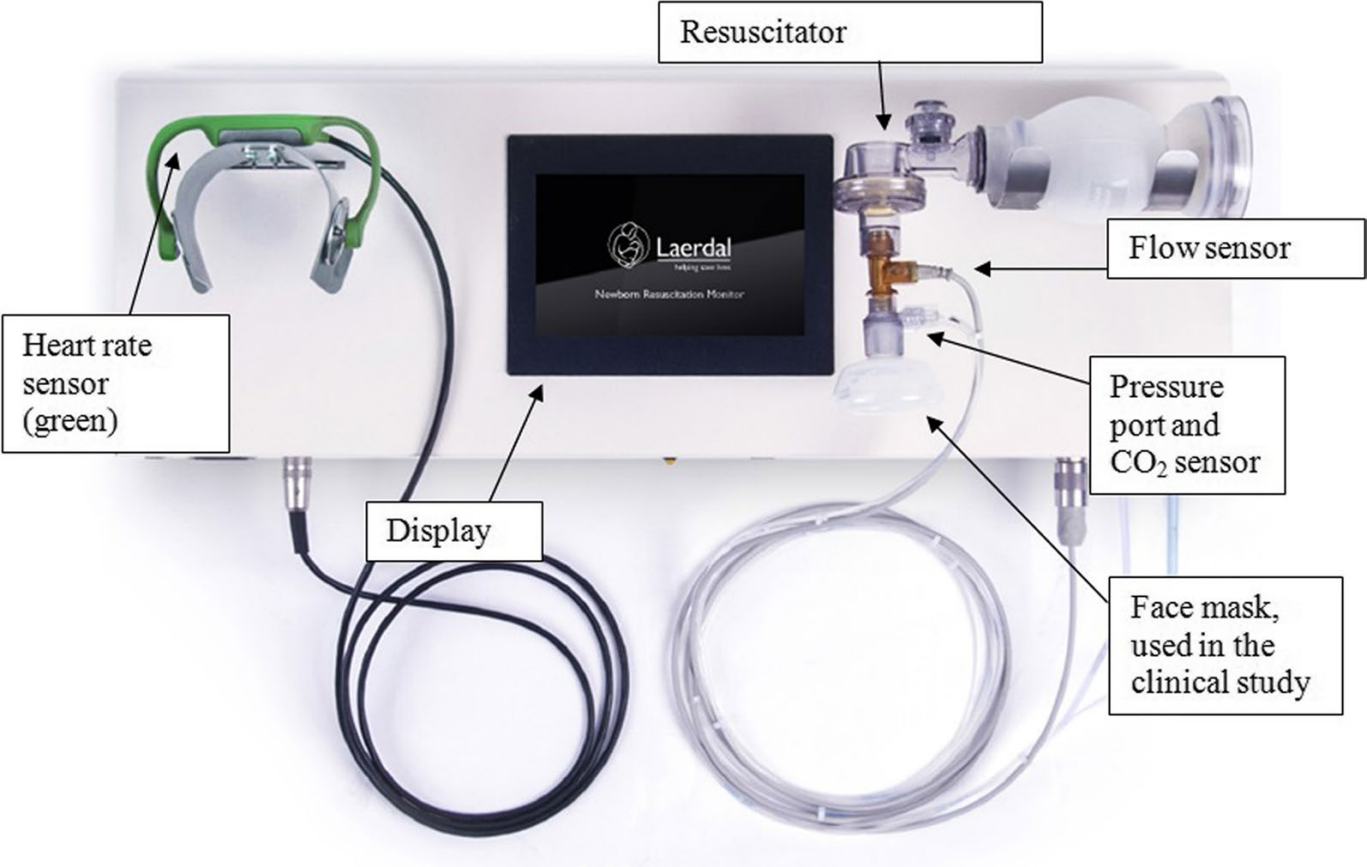
The newborn resuscitation monitor

HR is calculated from ECG measured by dry electrodes: Two stainless steel discs are mounted on each side of a flexible arch that is placed gently over thorax or abdomen of the subject (Fig. 2). The pressure exerted by the flexible arch is comparable to that of a stethoscope used for auscultation. The ECG is recorded and HR calculated using an proprietary algorithm based on a zero crossing count algorithm [12 **]**. The dry-electrode ECG technology and HR algorithm was validated (by the manufacturer) on adult human subjects (n = 30, 396 data points) against HR from a Philips HeartStart MRx (Philips Healthcare, Andover, MA) and showed a rate equivalence within 1 bpm in 84.3% of the data points, 99.8% within 4 bpm. The HR sensor is connected to the main cabinet through a cable, and the HR is displayed on the monitor.

The monitor thus uses reliable and proven technology put together into one unit. Safety for patient and operator is ensured by complying with current medical device technical standards, and is tested and approved for patient use according to the EU Medical Device Directive.

During the experiment, the HR sensor was placed over the abdomen of the piglet, and a Laerdal Neonatal Resuscitator size 1 (240 ml) was used for manual ventilations. Data from all sensors were stored in an internal memory card, downloaded to a computer through an USB connection, and analyzed using Matlab (MathWorks, Natick, MA).

### Protocol

The piglets were anesthetized in accordance with Norwegian guidelines for animal experiments. An ETT with cuff was inserted into the trachea and secured, and the HR sensor was placed over the abdomen. Anesthetic maintenance was provided. No interventions took place before the piglets were fully anesthetized. An intra-arterial line was established in one femoral artery to enable measurements of arterial pressures. The instrumented resuscitator (Fig. 2) was connected to the ETT, and baseline patterns and measurements of the inspiratory and expiratory pressure, volume, flow and EtCO_2_ at rest were recorded in the NRM. Each piglet underwent several experiments, and was euthanized with pentobarbital when mean arterial pressure decreased to ≤15 mbar.

Three experiments are described in this report; (1) measurements of EtCO_2_ with various tidal volumes, ventilation rates and dead space, and (2) blood was drawn from the arterial catheter for blood gas analysis and comparison of PaCO_2_ with EtCO_2_ every 4th minute. EtCO_2_ was recorded continuously by the monitor. (3) Lavage of surfactant. See Table 1 for description of the timeline for each piglet.

**Table 1:** Overview: overview of experiments and ventilations performed on each piglet

Type of experiment	Duration (min)	Dead space (ml)	Volume (ml/kg)	Rate (vpm)
EtCO_2_ measurements	4	0	10	40
	4	20	10	40
	4	10	10	40
	4	30	10	40
	1	0	10	20
	1	0	10	40
	1	0	6	40
	1	0	10	40
	1	0	15	40
	1	20	10	40
	1	20	10	20
	1	20	10	40
	1	20	6	40
	1	20	15	40
Pause		20	10	40
Surfactant deficiency	Lavage of the lungs through the ETT with 10 ml NaCl ten times in each series			
	Repeated lavage as long as mean arterial pressure >15			
	Ventilations with 100% O_2_, volume: 10 ml/kg, 40 ventilations per minute			

In the first part, EtCO_2_ was measured with different duration, dead space, volume and rates of inflation. Thereafter surfactant were lavaged out with 10 ml NaCl × 10 in each series and compliance and HR was measured after each series

Ventilations in room air were performed with different tidal volumes and ventilation rates (Table 2). Different dead space (0–30 ml) were provided and controlled by an expandable syringe connected between the tube and airway sensors. Each combination of volume and rate were kept for 1 min, whereas each dead space series lasted 4 min. EtCO_2_ was recorded continuously by the monitor.

**Table 2:** Experiment with different dead space and volumes: levels of EtCO_2_ for different tidal volumes and dead space, and then levels of EtCO_2_ with different rates of inflation per minute (ipm) and dead space

Volume (ml/kg)	EtCO_2_ (mmHg) 0 ml dead space^a^	EtCO_2_ (mmHg) 20 ml dead space^a^	p value
6	7.0 (5.3–7.8)	5.2 (4.3–6.0)	0.066
10	5.9 (4.8–7.2)	5.0 (4.2–6.2)	0.068
15	4.6 (4.1–5.9)	5.1 (3.6–5.5)	0.465

Dead space (ml)	EtCO_2_ at rate 20 (ipm)	EtCO_2_ at rate 40 (ipm)	p value
0	6.5 (5.2–7.4)	6.1 (4.5–7.0)	0.144
20	5.2 (3.4–6.2)	5.1 (4.2–6.2)	0.655

^a^Results are given as median (range)

During the surfactant deficiency experiment, the piglets were ventilated using 100% Oxygen, 40 ventilations/min and a volume of 10 ml/kg. We measured dynamic compliance [total expired volume/(peak inspiratory pressure − positive end expiratory pressure)]. Surfactant was lavaged from the piglet’s lungs with 10 ml saline ten times in each series (=100 ml in one series). Between each series of lavage, the piglets were ventilated for 3 min to allow steady state to occur (i.e. HR, ventilation pressure, and volume stabilizing at a certain level). The HR was registered continuously during the whole experiment. The series were repeated until the mean arterial pressure was ≤15 mbar.

### Data analysis

Difference between groups were analyzed by Wilkoxon signed rank test and results given as median (range). Changes in compliance during surfactant lavage were analyzed using a linear mixed model. A two sided *p* value of <0.05 was considered to be statistically significant. The levels of EtCO_2_ and PaCO_2_ and the difference in compliance was compared between groups by Wilkoxon signed rank test. A p value of <0.05 was considered to be statistically significant. Data were analyzed using the IBM-SPSS statistical package (IBM SPSS Statistics for Windows, Version 22.0. Armonk, NY: IBM Corp), and figures were made using Matlab R2014a (MathWorks, Natick, MA).

## Results

Data can be viewed in detail in availability of data and materials.

One piglet was excluded in both experiments due to excessive leakage around the endotracheal tube (ETT). The ventilation and HR signals were stored in the monitor correctly aligned in time, and data from each ventilation could be analyzed individually (Fig. 1).

### EtCO_2_ readings with different dead space, volumes and ventilation rates

Different clinical scenarios were simulated and Table 2 shows the levels of EtCO_2_ during different tidal volumes (inflated volumes) (6–15 ml/kg), dead space (different size of mask/tube in airway) (0 and 20 ml) and ventilation rates (the rate of inflations given) (20 and 40 ventilations per minute). There was a tendency of lower EtCO_2_ levels with increasing dead space, i.e. 0 ml versus 20 ml, for the two smallest tidal volumes, i.e. 6 and 10 ml/kg (p = 0.066 and p = 0.068, respectively). Ventilation rates of 20 compared to 40 ventilations per minute did not influence the levels of EtCO_2_ with 0 and 20 ml dead space.

EtCO_2_ and PaCO_2_ were comparable and stable with dead spaces of 10 and 20 ml However, at 30 ml dead space there was a tendency for lower EtCO_2_ compared to PaCO_2_ (Table 3).

**Table 3:** Dynamic compliance and HR before and after lavage of surfactant from the lungs

Piglet	Dynamic compliance before lavage (ml/mbar)	HR before lavage (bpm)	Number of lavage series (10 × 10 ml) performed	Dynamic compliance after lavage (ml/mbar)	HR after lavage (bpm)
B (4.7 kg)	6.6	149	9	3.5	138
C (5.2 kg)	4.3	144	13	2.5	143
D (5.2 kg)	2.7	136	12	1.5	193
E (3.7 kg)	8.0	132	3	2.0	132

Measurements from before lavage the first series (10 times 10 ml saline) and after the last lavage series performed

### Surfactant lavage

**Fig. 3:**
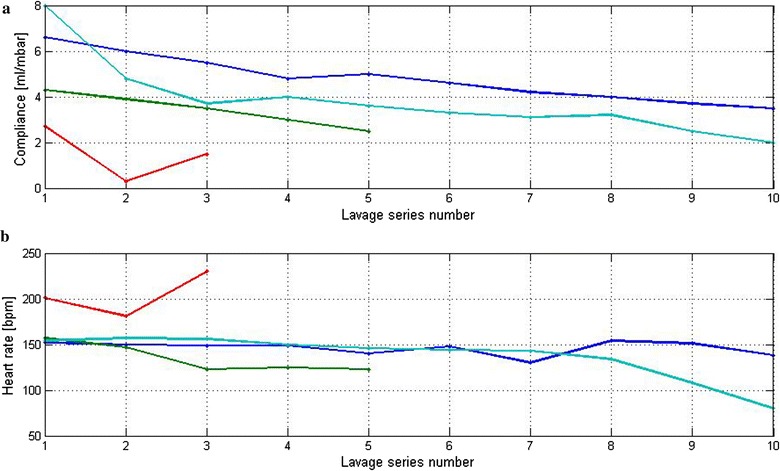
Lavage of surfactant: measured HR and dynamic compliance over time for piglets B (*green line*), C (*turquoise line*), D (*blue line*) and E (*red line*) during lavage of surfactant with 10 × 10 ml of saline. **a** shows the dynamic compliance measured at the corresponding times. **b** shows HR measured after each series of 10 × 10 ml saline lavage

The dynamic compliance was measured in each piglet before the lavage of surfactant. The dynamic compliance and HR before and after the lavage series for each piglet are shown in Table 4. There was an average decrease in compliance by 3% after each lavage series (p = 0.004) (Fig. 3, panel a). The piglets tolerated 3–13 series of lavage before the mean arterial pressure decreased to ≤15 mbar. Piglet E with the lowest body weight tolerated three lavage series until mean arterial pressure was ≤15 mbar. ECG was continuously recorded and successfully stored during all the experiments. The HR remained stable throughout the lavage (Fig. 3, panel b).

**Table 4:** Levels of EtCO_2_ and PaCO_2_ measured every 4 min with different dead space

Dead space (ml)	EtCO_2_ (mmHg)^a^	PaCO_2_ (mmHg)^a^	p value
0	7.1 (5.0–9.7)	7.3 (5.0–9.2)	0.502
10	6.7 (5.2–8.0)	6.7 (5.0–7.7)	0.724
20	6.7 (5.0–7.7)	6.3 (4.9–8.4)	0.141
30	5.0 (3.9–5.6)	7.3 (5.4–8.4)	0.075

Ventilation volume was 10 ml/kg and ventilation rate was 40 ventilations per minute

^a^Results are given as median (range)

## Discussion

The monitor has the ability to continuously display HR using dry electrode ECG technology, to measure tidal volume, pressure and EtCO_2_ and to store the results for later analysis. The planned clinical studies will hopefully provide new knowledge on neonatal transition and resuscitation; about ventilation pressure and volume and thereby compliance, as well as mask leakage, EtCO_2_, and HR during face mask ventilation. The NRM will be used to study if measurements and feedback to the provider will facilitate more objective assessments and thus improve decision making and quality of care during newborn resuscitation.

Accuracy level of volumes and pressures registered during the controlled ventilations was in accordance with our expectations. We further observed a steady and significant decrease in compliance when surfactant was lavaged from the piglets lungs. The continuous fall in compliance of mean 3% for each lavage, underlines the monitors ability to detect minute changes in volume and pressure over time. This observed ability of the NRM to register physiological signal data is reassuring.

Previous findings suggest that EtCO_2_ levels can indicate lung aeration, but is not an accurate measure of PaCO_2_ [4]. Our findings were in line with these previous findings although we had a small number of measurements. It seemed EtCO_2_ was varying according to dilution of expired gas in terms of increased dead space and change in tidal volumes. This, as well as the delays in side stream technology, is important to consider when moving to clinical studies, and suggests EtCO_2_ may only be useful as an indication of presence or change in aeration in the setting of bag/mask ventilation.

The dry electrode ECG sensor detected satisfactory ECG signals throughout the study. In the clinical setting, the skin of newborns is likely to be moister which would further increase the dry electrodes’ capability of detecting ECG signals. Importantly, HR is the recommended indicator of successful ventilations [13], and the dry electrode ECG technique can provide valuable real-time feedback to the provider. Validation to conventional ECG was satisfactory. However, it was performed on adults and it precision and accuracy in newborns is yet to be studied.

## Conclusions

The prototype NRM was capable of continuously displaying HR and detecting inflicted changes in ventilation and compliance of piglets. It can record parameters such as given volume and pressure and the responses (changes in HR and EtCO_2_). This monitor may facilitate research during neonatal transition and resuscitation that can provide valuable new insight.

## Abbreviations

NRM: newborn resuscitation monitor
HR: heart rate
EtCO_2_: end tidal carbon dioxide
PPV: positive pressure ventilation
ETT: endotracheal tube
mbar: millibar
